# Evaluating a new partner management strategy for bacterial sexually transmitted infections after a change in 2020 in Amsterdam, the Netherlands, 2017 to 2023

**DOI:** 10.2807/1560-7917.ES.2026.31.22.2500754

**Published:** 2026-06-04

**Authors:** Buhari Teker, Maarten F Schim van der Loeff, Lois Deden, Jason Schouten, Elske Hoornenborg, Vita W Jongen, Henry JC de Vries

**Affiliations:** 1 Department of Infectious Diseases, Public Health Service of Amsterdam (GGD Amsterdam), Amsterdam, the Netherlands; 2 Department of Dermatology, Amsterdam UMC location University of Amsterdam, Amsterdam, The Netherlands; 3 Department of Internal Medicine, Amsterdam UMC location University of Amsterdam, Amsterdam, The Netherlands; 4 Amsterdam Institute for Immunology & Infectious Diseases (AI&I), Amsterdam, the Netherlands; 5 Amsterdam Public Health research institute (APH), Amsterdam, the Netherlands; 6 Stichting hiv monitoring, Amsterdam, the Netherlands

**Keywords:** *Neisseria gonorrhoeae*, *Chlamydia trachomatis*, Sexually Transmitted Diseases, Bacterial, Contact Tracing, Sexual Partners, male, women, Heterosexuality, Homosexuality, Sexual and Gender Minorities

## Abstract

**BACKGROUND:**

From 1 March 2020, the Centre for Sexual Health Amsterdam limits presumptive treatment for chlamydia or gonorrhoea to clients notified by steady partners. Treatment after notification by non-steady partners requires a positive test result.

**AIM:**

We aimed to evaluate this change in policy on unnecessary antibiotic treatment, time to treatment and lost to follow-up clients.

**METHODS:**

We included consultations (with testing) following partner notification for chlamydia or gonorrhoea between 1 March 2017 and 1 March 2023. Primary outcome was unnecessary antibiotic treatment (presumptive treatment with negative test result) before vs after the policy change. We also assessed return for treatment of confirmed and not presumptively treated individuals after the policy change.

**RESULTS:**

From 4,579 consultations, unnecessary antibiotics were prescribed for 786/1,318 (59.6%) clients for chlamydia and 729/1,117 (65.3%) clients for gonorrhoea before 1 March 2020. After 1 March 2020, this decreased to 324/1,275 (25%) and 341/1,369 (25%), respectively, corresponding to a relative reduction of 58% for chlamydia (adjusted relative risk (aRR): 0.42; 95% confidence interval (CI): 0.37–0.47) and 63% (aRR: 0.37; 95% CI: 0.33–0.42) for gonorrhoea. After the policy change, clients not given presumptive treatment but later diagnosed returned after a median of 7 days (interquartile range (IQR): 5–8) for chlamydia and 6 days (IQR: 4–7) for gonorrhoea. Missed treatment occurred in 4/155 (2.6%) of chlamydia and 5/158 (3.2%) of gonorrhoea consultations.

**CONCLUSION:**

The new partner management protocol substantially reduced unnecessary antibiotic treatment for chlamydia and gonorrhoea. The majority of confirmed infections still received appropriate treatment.

**Key public health message**

**What did you want to address in this study and why?**

We evaluated the impact of a policy change - limiting presumptive antibiotic treatment for chlamydia and gonorrhoea to clients notified by steady partners - on unnecessary treatment.

**What have we learnt from this study?**

We found that the policy change substantially reduced unnecessary antibiotic treatment while maintaining high follow-up for confirmed infections in clients who did not receive presumptive treatment.

**What are the implications of your findings for public health?**

The impact of the policy change underscores the importance of adapting treatment protocols to reduce unnecessary antibiotic treatment, while ensuring effective care for individuals with chlamydia and/or gonorrhoea.

## Introduction

Partner notification and presumptive partner treatment are strategies to reduce onward transmission and reinfection of bacterial sexually transmitted infections (STIs) [1]. This involves informing recent sexual partners of an individual with a confirmed STI (i.e. the index case) about potential exposure to an STI, referring them for STI testing and, if indicated, presumptive treatment administration before test results are available. Presumptive treatment is provided when an infection is likely, based on confirmed recent exposure, clinical presentation or preliminary laboratory results. Delaying the treatment of sexual partners poses a risk of loss to follow-up, and, potentially, onward transmission [2,3]. Studies show that presumptive partner treatment reduces persistent infections and reinfection of *Chlamydia trachomatis* (Ct) and *Neisseria gonorrhoeae* (Ng) and may even lower the incidence of Ct and Ng at population level [4,5].

However, presumptive partner treatment also increases antibiotic consumption, contributing to the development of antimicrobial resistance (AMR) [6,7]. Treatment failure with first-line treatment for Ct (i.e. oral azithromycin or doxycycline) is rare globally [8]. However, reduced susceptibility to ceftriaxone in Ng has been detected in the Netherlands since 2020, and ceftriaxone-resistant Ng strains have been identified in other European countries [9,10]. Moreover, any administered antibiotic can induce AMR in other pathogens that the individual is harbouring and has a potentially adverse impact on the microbiome [11].

International guidance on presumptive partner treatment is not uniform [12-17]. In the Netherlands, the Centre for Sexual Health (CSH) Amsterdam changed its policy on partner management on 1 March 2020 in an effort to reduce unnecessary antibiotic treatment [18,19]. Whereas previously all formally notified clients received presumptive treatment, the revised policy introduces a more selective approach to presumptive partner treatment.

Given the need to balance reducing unnecessary antibiotic treatment with the risk of delayed treatment and loss to follow-up, we evaluated the impact of this policy change in presumptive partner treatment for Ct and Ng among asymptomatic clients. Specifically, we addressed two endpoints: (i) unnecessary antibiotic treatment, defined as presumptive antibiotic treatments given to clients who subsequently tested negative for Ct or Ng before and after the policy change, and (ii) time to treatment and loss to follow-up among those who tested positive for Ct or Ng but did not receive presumptive treatment after the policy change.

## Methods

### Study design and participants

The Centre for Sexual Health Amsterdam provides free of charge STI-related and sexual healthcare for all clients under 25 years of age and also for other clients at increased risk of STIs regardless of their age (e.g. those notified of an STI, men who have sex with men (MSM), sex workers and those presenting with symptoms) as described previously [20]. We analysed routinely collected data from individuals tested for bacterial STIs between 1 March 2017 and 1 March 2023 (i.e. 3 years before and 3 years after the policy change in presumptive partner treatment for Ct and Ng).

We included data of all asymptomatic clients with a formal partner notification for Ct and/or Ng (i.e. those notified by an index case with a Ct and/or Ng laboratory-confirmed diagnosis, with the notification verified by a healthcare provider) and excluded those with an informal partner notification (i.e. notified by an index case without the involvement of a healthcare professional). Presumptive treatment was previously not indicated for informally notified partners and therefore clients with informal partner notifications were unaffected by the policy change. Clients presenting with symptoms suggestive of an STI are always treated presumptively according to standard practice and were therefore also excluded from this analysis. The study population thus consists of asymptomatic clients who attended CSH Amsterdam after a formal partner notification, for whom treatment decisions were affected by the policy change in presumptive treatment.

Some clients visited CSH Amsterdam more than once during the study period. For this reason, the number of consultations exceeds the number of clients. This study used routinely collected, anonymised data from CSH Amsterdam. As data were obtained as part of standard care, individual informed consent was not required.

### Partner management

Partner management at CSH Amsterdam aims to prevent STI transmission and reinfection by tracing, notifying, testing and, if necessary, treating sexual partners of index cases with an STI. For asymptomatic consenting index clients diagnosed with Ct and/or Ng, we notified all sexual partners within the preceding 6 months, and for symptomatic index clients all sexual partners within the preceding 6 weeks. We recommended that notified partners visit CSH Amsterdam for STI testing and further care. Of note, clients with a formal partner notification are always tested for Ct and Ng, but treatment may be presumptively prescribed or prescribed only after test results are available.

#### Policy for presumptive treatment for *Chlamydia trachomatis* and *Neisseria gonorrhoeae* before 1 March 2020

Before 1 March 2020, presumptive treatment was offered to all formally notified clients without awaiting test results (Supplementary Figure S1 shows a flowchart of the partner management policies before and after 1 March 2020 in partner notification consultations for *Chlamydia trachomatis* and *Neisseria gonorrhoeae*). Presumptive treatment was always provided to clients notified for lymphogranuloma venereum (LGV).

#### Policy for presumptive treatment for *Chlamydia trachomatis* and *Neisseria gonorrhoeae* after 1 March 2020

After 1 March 2020, presumptive treatment was offered only to formally notified sexual partners: (i) who had sex with the index case in the previous 2 months, and (ii) intended to have sex with the index again (steady partnership). All other formally notified partners received treatment only after a positive test result.

All clients with a positive test result who had not yet received treatment were sent up to three reminders every 2 weeks (via email, phone or text). Notified clients who tested positive for Ct or Ng but did not receive presumptive treatment or respond to reminders were considered lost to follow-up if they had not visited the clinic for treatment within 60 days after their test result became available. We excluded clients who were eligible but declined presumptive treatment, as well as those not eligible but treated presumptively at the physician’s discretion.

### Procedures

The general testing procedures for CSH Amsterdam have been described previously [20]. The anatomical locations sampled during the entire study period for MSM, men who have sex with women (MSW), women and transgender and gender diverse people are summarised in Table 1. All diagnoses of Ct and Ng were detected by nucleic acid amplification testing (NAAT). Treatment was provided free of charge and included a single 1 g dose of oral azithromycin for oral/urogenital Ct and oral doxycycline 100 mg twice daily for 7 days for anorectal Ct. A single 1 g dose of intramuscular ceftriaxone was offered for Ng. These regimens were used both for presumptive treatment and for treatment after confirmed infection. Routinely collected demographic data (e.g. age and gender), clinical data (e.g. HIV status and HIV pre-exposure prophylaxis (PrEP) use) and information on sexual behaviour (e.g. sexual preference and number of sexual partners) were extracted from electronical medical records. Multiple infections with the same bacterium at different anatomical locations on the same date were recorded as one single infection of that bacterium.

**Table 1:** Anatomical locations tested for *Chlamydia trachomatis* and *Neisseria gonorrhoeae* by subpopulation, the Netherlands, 2017–2023

Anatomical location	MSM/MSMW	MSW	Women	Transgender and gender diverse people
Pharynx	Yes	No	Yes^b^	Yes
Anus	Yes	No	Yes^b^	Yes^b^
Urethra^a^	Yes	Yes	No	Yes^b^
Vagina/cervix	No	No	Yes	Yes^b^

MSM: men who have sex with men; MSMW: men who have sex with men and women; MSW: men who have sex with women.

^a^ Urine sample from penile urethra.

^b^ Depending on clinical and sexual behaviour history.

### Outcomes

The primary outcome was unnecessary antibiotic treatment before and after the policy change. Unnecessary antibiotic treatment was assessed for each consultation individually and dichotomised as yes (presumptive treatment provided but negative NAAT test result) or no (presumptive treatment provided and positive NAAT test result).

Two secondary outcomes were assessed during the new policy period only among clients who did not receive presumptive treatment: (i) time to treatment, defined as the number of days between the initial consultation and the treatment consultation (continuous variable), and (ii) the proportion of missed treatments (lost to follow-up), defined as the absence of treatment within 60 days after the test result was known (binary: no/yes).

### Covariates

The following client characteristics were included as covariates. Gender and sexual preference were categorised as MSM, MSW, women, or transgender and gender diverse people. Age was grouped into four categories (< 25, 25–29, 30–34 and ≥ 35 years). Country of birth was classified as the Netherlands or other and education level as none, primary school, secondary school, other or college/university. HIV status and oral HIV PrEP use were combined into three categories: HIV-negative without HIV PrEP use, HIV-negative with HIV PrEP use and living with HIV. History of bacterial STI was included as a binary variable (no/yes). The number of sexual partners was analysed as a continuous variable.

### Statistical analysis

We described sociodemographic, clinical and behavioural characteristics measured at the first consultation of each client who attended CSH Amsterdam following partner notification for Ct and/or Ng. Characteristics were described before and after the policy change and compared using Pearson’s chi-square or Fisher’s exact test for categorical variables, and Student’s t-test or Wilcoxon rank-sum test for continuous variables.

As the revised policy applied only to clients with formal notifications by a steady partner, we also examined whether the distribution of steady vs non-steady formal partner notifications changed between the two periods. To estimate changes in unnecessary antibiotic treatment after the policy change compared with before, we applied relative risk (RR) regression (i.e. generalised estimating equations with a Poisson distribution, log link and robust variance estimation) to account for repeated consultations (i.e. multiple consultations per client). An exchangeable working correlation structure was specified; results were similar when assuming an independent correlation structure. Results are presented as RR with 95% confidence intervals (CIs).

Analyses were performed separately for Ct and Ng. Clients with both a Ct and Ng infection were included in both analyses. In the multivariable models, we adjusted for client characteristics that differed significantly between the two time periods in the aforementioned descriptive analysis. Variables that differed between the periods were considered potential covariates in subsequent regression analyses as they may reflect changes over time in the client population attending CSH Amsterdam after a partner notification and could be associated with infection probability and healthcare consumption. For categorical variables with more than 100 missing values, a separate ‘missing’ category was created. In addition, we performed a complete-case analysis (whereby we excluded all records with missing values) to assess the effect of including a ‘missing’ category on the outcome. Results are presented as crude and adjusted RRs (aRR) with 95% CIs. To account for temporal trends coinciding with the COVID-19 pandemic, we included a sensitivity analysis where we additionally adjusted for calendar time.

Time to treatment was analysed using Kaplan-Meier methods at the consultation level, with receipt of antibiotic treatment (azithromycin or doxycycline for Ct, ceftriaxone for Ng) as the event. Clients who had not returned for treatment within 60 days of their test result becoming available were censored and classified as having missed treatment.

A sensitivity analysis was performed for the primary outcome (i.e. the change in unnecessary antibiotic treatment after the policy change) to assess whether the observed effect differed across subgroups. This analysis aimed to explore potential effect modification and to evaluate the robustness of the findings across demographic groups. We stratified the analysis by gender and sexual behaviour (MSM, MSW and women) and by age group (< 25, 25–29, 30–34 and ≥ 35 years). The subgroup of transgender and gender diverse people was not included due to the small sample size.

We considered a p value of < 0.05 as statistically significant. Statistical analyses were performed in Stata version 17 (StataCorp, College Station, United States (US)).

## Results

**Figure 1:**
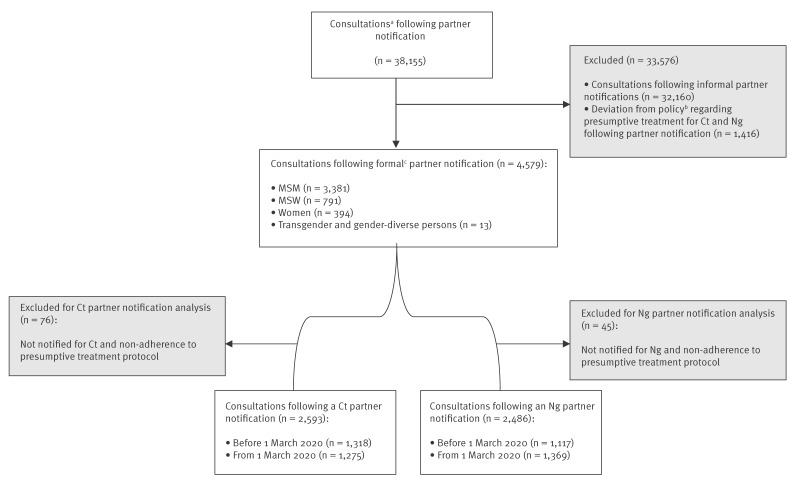
Inclusion flowchart to compare a presumptive partner treatment policy change for *Chlamydia trachomatis* and *Neisseria gonorrhoeae*, Centre for Sexual Health, Amsterdam, the Netherlands, 1 March 2017–1 March 2023

Between 1 March 2017 and 1 March 2023, there were 38,155 consultations following partner notification for Ct and/or Ng. Of these consultations, 32,160 (84.3%) involved informal partner notifications and in 1,416 (3.7%) presumptive treatment was either declined despite eligibility (n = 622) or provided outside protocol at the physician’s discretion (n = 794); both these groups were excluded from the analysis (Figure 1). Presumptive treatment was more often declined before the policy change (n = 490, 85%) compared with under the new policy (n = 132, 16%), while presumptive treatment provision at the physician’s discretion was more common under the new policy (n = 703, 84%) compared with the old (n = 91, 16%). Thus, 4,579 consultations among 3,701 clients were included in the analysis, of which 3,381 (73.8%) involved MSM, 791 (17.3%) MSW, 394 (8.6%) women, and 13 (0.3%) transgender and gender diverse persons.

Ct: *Chlamydia trachomatis*; Ng: *Neisseria gonorrhoeae;* MSM: men who have sex with men; MSW: men who have sex with women.

^a^ Consultations could include notifications for both Ct and Ng; therefore, the total number of consultations before and after 1 March 2020 does not equal the sum of those notified for Ct and Ng.

^b^ Deviation from policy' refers to notified clients who were eligible for presumptive treatment but declined it, as well as those who were not eligible for presumptive treatment but received it at the physician’s discretion.

^c^ A formal notification refers to approval by a healthcare professional based on a laboratory-confirmed diagnosis in the index case. An informal notification refers to notifying someone without the involvement of a healthcare professional.

Of the 3,701 included clients, 3,118 (84.3%) had one consultation, 423 (11.4%) had two consultations and 160 (4.3%) had three or more consultations during the study period. Socio-demographic and behavioural characteristics of clients included in the analysis from the initial consultation are presented in Table 2. Changes in the type of partners notified following the policy change are summarised in Table 3: under the new policy, partner notifications more often involved steady partners (57.8% vs 38.3%; RR: 1.48; p < 0.001) and less often non-steady partners (43.3% vs 62.3%; RR: 0.71; p < 0.001).

**Table 2:** Sociodemographic and behavioural characteristics of formally notified clients at first consultation, Centre for Sexual Health, Amsterdam, the Netherlands, 1 March 2017–1 March 2023

Characteristics	Total (n = 3,701)		Before 1 March 2020 (n = 1,931)		After 1 March 2020 (n = 1,770)		p value
	n	%	n	%	n	%	
Gender and sexual preference, categorised							
MSM	2,535	68.5	1,220	63.2	1,315	74.3	< 0.001
MSW	763	20.6	482	25.0	281	15.9	
Women	390	10.5	226	11.7	164	9.3	
Transgender and gender diverse people	13	0.4	3	0.2	10	0.6	
Age (years)							
Median (IQR)	30 (24–38)		29 (24–37)		31 (25–38)		< 0.001
< 25	960	25.9	550	28.5	410	23.2	< 0.001
25–29	839	22.7	453	23.5	386	21.8	
30–34	660	17.8	313	16.2	347	19.6	
≥ 35	1,242	33.6	615	31.8	627	35.4	
Born in the Netherlands							
Yes	2,326	62.8	1,300	67.3	1,026	58.0	< 0.001
No	1,375	37.2	631	32.7	744	42.0	
Highest education level							
None, primary, secondary school or other	1,207	33.1	716	37.6	491	28.2	< 0.001
College/university	2,439	66.9	1,189	62.4	1,250	71.8	
Number of sexual partner(s)^a^							
Median (IQR)	5 (2–10)		5 (2–10)		5 (3–10)		0.006
HIV status and oral HIV PrEP use^a^							
HIV negative and no oral HIV PrEP use	2,450	66.2	1,527	79.1	923	52.1	< 0.001
HIV negative and oral HIV PrEP use	693	18.7	81	4.2	612	34.6	
Living with HIV	558	15.1	323	16.7	235	13.3	
History of bacterial STI^b,c^							
No	1,270	50.3	656	52.6	614	48.1	0.023
Yes	1,254	49.7	591	47.4	663	51.9	

Ct: *Chlamydia trachomatis*; IQR: interquartile range; MSM: men who have sex with men; MSW: men who have sex with women; Ng: *Neisseria gonorrhoeae*; PrEP: pre-exposure prophylaxis; STI: sexually transmitted infection.

^a^ In the 6 months before the initial consultation.

^b^ In the 12 months before the initial consultation (self-reported).

^c^ Includes gonorrhoea, chlamydia, lymphogranuloma venereum and syphilis.

p values were calculated using Pearson’s chi-square test or Fisher’s exact test (for categorical data) and Student’s t-test or Wilcoxon rank-sum test (for continuous data).

Data were missing for: highest education level (n = 28 and n = 27), number of sexual partner(s) (n = 14/n = 12) and history of bacterial STI (n = 703 and n = 434) for before 1 March 2020 and from 1 March 2020, respectively.

**Table 3:** Formal partner notification consultations by partner type, Centre for Sexual Health, Amsterdam, the Netherlands, 1 March 2017–1 March 2023

Partner type	Total number of consultations (n = 4,579)		Consultations before 1 March 2020 (n = 2,204)		Consultations after 1 March 2020 (n = 1,917)		RR		p value
	n	%	n	%	n	%	RR	95% CI	
Partner notification by steady partner(s)									
No	2,171	52.7	1,361	61.7	810	42.3	Reference		< 0.001
Yes	1,950	47.3	843	38.3	1,107	57.7	1.48	1.38–1.58	
Missing	458	NA	25	NA	433	NA	NA		NA
Partner notification by non-steady partner(s)									
No	1,917	46.5	830	37.7	1,087	56.7	Reference		< 0.001
Yes	2,204	53.5	1,374	62.3	830	43.4	0.71	0.66–0.75	
Missing	458	NA	25	NA	433	NA	NA		NA

CI: confidence interval; Ct: *Chlamydia trachomatis*; NA: not applicable; Ng: *Neisseria gonorrhoeae*; RR: relative risk.

A steady partner is a notified client who: (i) had sex with the index client in the previous 2 months, and (ii) intents to have sex with the index client again; non-steady partnership are all other sexual engagements.

p values were derived from RR regression (generalised estimating equations with a Poisson distribution, log link, and robust variance estimation) to account for repeat consultations per client.

### Changes in unnecessary antibiotic treatment

#### Partner notifications for *Chlamydia trachomatis*

**Figure 2:**
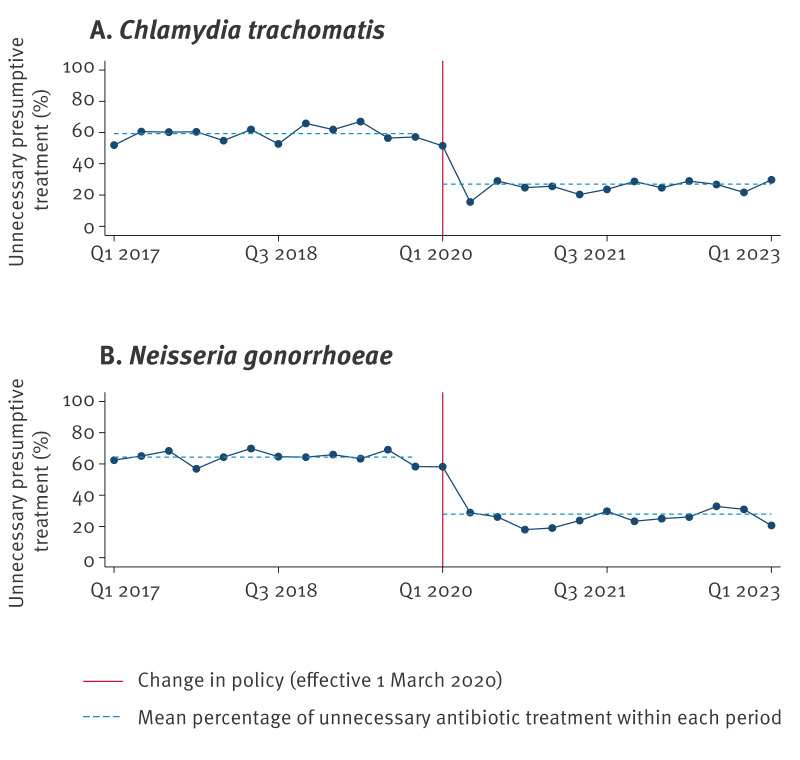
Quarterly proportion of presumptive treatment resulting in unnecessary antibiotic treatment in partner notifications for (A) *Chlamydia trachomatis* and (B) *Neisseria gonorrhoeae*, Centre for Sexual Health, Amsterdam, the Netherlands, 1 March 2017–1 March 2023

For the Ct analysis, we additionally excluded 76 consultations: 58 consultations involved formal partner notification for Ng only (and no Ct partner notification) and in 18 consultations, no presumptive treatment was given due to unknown partnership. Before 1 March 2020, 786 of 1,318 (59.6%) consultations with presumptive treatment for Ct resulted in unnecessary antibiotic treatment (i.e. treatment was followed by a negative test result). The distribution of unnecessary treatment in Ct partner notifications over time is shown in Figure 2.

Q: quarter.

Clients with both a *Chlamydia trachomatis* and *Neisseria gonorrhoeae* infection were included in both analyses.

Under the new policy, from 1 March 2020, of the 1,275 consultations involving Ct partner notifications, 661 consultations did not involve presumptive treatment due to the policy change but would have been presumptively treated during the old protocol. The remaining 614 did receive presumptive treatment and 324 of these resulted in unnecessary antibiotic treatment. Thus, 25.4% (324/1,275) of consultations following formal partner notification resulted in unnecessary antibiotic treatment. This corresponds to a relative reduction of 58% in unnecessary antibiotic treatment under the new policy compared with the old policy (aRR: 0.42; 95% CI: 0.37–0.47; p < 0.001) (Table 4). Adjusting for potential temporal trends coinciding with the COVID-19 pandemic resulted in a slightly higher reduction in unnecessary antibiotic treatment (aRR: 0.39; 95% CI: 0.32–0.49).

**Table 4:** Change in unnecessary antibiotic treatment given during consultations following formal partner notification for *Chlamydia trachomatis* or *Neisseria gonorrhoeae* infections, Centre for Sexual Health, Amsterdam, the Netherlands, 1 March 2017–1 March 2023

Consultations	Unnecessary antibiotic treatment number of treatments		Unnecessary antibiotic treatment univariable RR regression			Unnecessary antibiotic treatment multivariable RR regression		
	n / N	%^b^	Crude RR	95% CI	p value^c^	aRR^d^	95% CI	p value^c^
All consultations following *Chlamydia trachomatis* partner notifications								
**Policy** ^a^								
Old	786 / 1,318	59.6	Reference			Reference		
New	324 / 1,275	25.4	0.43	0.38–0.47	< 0.001	0.42	0.37–0.47	< 0.001
All consultations following *Neisseria gonorrhoeae* partner notifications								
**Policy** ^a^								
Old	729 / 1,117	65.3	Reference			Reference		
New	341 / 1,369	24.9	0.38	0.34–0.42	< 0.001	0.37	0.33–0.42	< 0.001

aRR: adjusted relative risk; CI: confidence interval; RR: relative risk.

^a^ The old policy refers to the period before 1 March 2020, whereas the new policy refers to the period from 1 March 2020.

^b^ The numerator is the number of consultations where presumptive treatment was prescribed unnecessarily (i.e. unnecessary antibiotic treatment). The denominator is the total number of consultations with *Chlamydia trachomatis* (Ct) or *Neisseria gonorrhoeae* (Ng) partner notification.

^c^ P values were derived from RR regression (generalised estimating equations with a Poisson distribution, log link and robust variance estimation) to account for repeated consultations per individual.

^d^ Adjusted for gender and sexual behaviour, age, country of birth, highest education level, number of sexual partners in the last 6 months, HIV status and HIV pre-exposure prophylaxis use and self-reported history of a sexually transmitted infection (STI) in the last 6 months. Values were missing for highest education level (n = 35 for Ct, n = 31 for Ng), number of sexual partners (n = 22 for Ct, n = 21 for Ng), and self-reported history of an STI (n = 889 for Ct, n = 465 for Ng). We categorised the missing values in self-reported history of an STI as a separate category when more than 100 records had missing values. A complete-case analysis (where all missing values were removed including those from self-reported history of an STI) resulted in an aRR of 0.39 (95% CI 0.33–0.45) for Ct and 0.37 (95% CI: 0.32–0.42) for Ng.

Analysis stratified by gender and sexual behaviour showed a substantial reduction of unnecessary antibiotic treatment under the new policy across all groups: MSM (aRR: 0.35; 95% CI: 0.30–0.40; p < 0.001), MSW (aRR: 0.61; 95% CI: 0.50–0.75; p < 0.001) and women (aRR: 0.46; 95% CI: 0.31–0.68; p < 0.001) (Supplementary Table S1 presents sensitivity analyses of the change in unnecessary antibiotic use in partner notification consultations for *Chlamydia trachomatis*, stratified by gender, sexual behaviour, and age group). Analysis stratified by age group showed consistently lower risks under the new policy, with statistically significant reductions in all age categories (aRR ranging from 0.35 to 0.46; all p < 0.001).

#### Partner notifications for *Neisseria gonorrhoeae*

For the Ng analysis, we additionally excluded 45 consultations: 35 involved formal partner notification of Ct only (and no Ng partner notification) and in 10 consultations, no presumptive treatment was given due to unknown partnership. Before 1 March 2020, 729 of 1,117 (65.3%) consultations with presumptive treatment for Ng resulted in unnecessary antibiotic treatment. The distribution of unnecessary treatment in Ng partner notifications over time is shown in Figure 2.

Under the new policy, from 1 March 2020, of the 1,369 consultations involving Ng partner notifications, 778 consultations did not involve presumptive treatment due to the policy change but would have been presumptively treated in the old protocol. The remaining 591 did involve presumptive treatment and of these, 341 resulted in unnecessary antibiotic treatment. Thus 24.9% (341/1,369) consultations following formal partner notification resulted in unnecessary antibiotic treatment. This corresponds to a relative reduction of 63% in unnecessary antibiotic treatment under the new policy compared with the old policy (aRR: 0.37; 95% CI: 0.33–0.42; p < 0.001). Adjusting for potential temporal trends coinciding with the COVID-19 pandemic resulted in a similar reduction in unnecessary antibiotic treatment (aRR: 0.38; 95% CI: 0.31–0.46).

Analysis stratified by gender and sexual behaviour showed a strong reduction in unnecessary antibiotic treatment under the new policy among MSM (aRR: 0.37; 95% CI: 0.32–0.41; p < 0.001), MSW (aRR: 0.39; 95% CI: 0.24–0.64; p < 0.001) and women (aRR: 0.43; 95% CI: 0.20–0.95; p = 0.036) (Supplementary Table S2 presents sensitivity analyses of the change in unnecessary antibiotic use in partner notification consultations for *Neisseria gonorrhoeae*, stratified by gender, sexual behaviour, and age group). Analysis stratified by age group also showed significant reductions across all groups (aRR ranging from 0.29 to 0.40; all p < 0.001).

### Treatment delay and missed treatments during the new policy period

During the new policy period, in consultations with clients notified for Ct who were not presumptively treated (n = 661), 155 (23.5%) clients had a positive NAAT result for Ct. For 20 consultations, the time until return to the clinic was unknown. For the remaining 135 consultations, the median time to return for treatment to clinic was 7 days (IQR: 5–8; range: 2–53).

**Figure 3:**
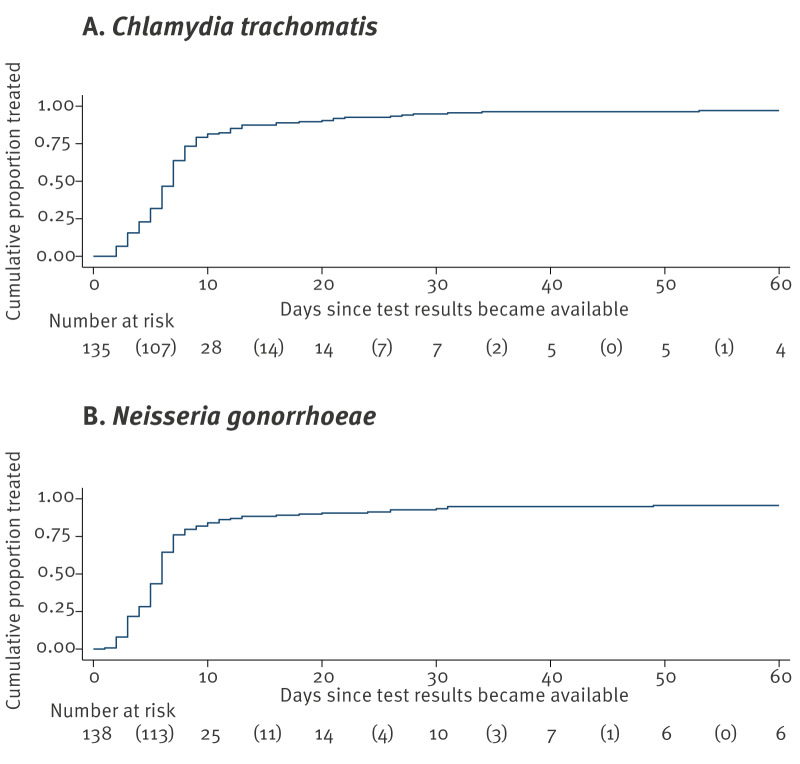
Kaplan–Meier estimates of time to treatment during the new policy period among clients with (A) *Chlamydia trachomatis* or (B) *Neisseria gonorrhoeae* infection who were not presumptively treated, Centre for Sexual Health, Amsterdam, the Netherlands, 1 March 2020–1 March 2023

In consultations with clients notified for Ng who were not presumptively treated (n = 778), 158 (20.3%) clients had a positive NAAT result for Ng. For 20 consultations, the time until return to the clinic was unknown. For the remaining 138 consultations, the median time to return to the clinic was 6 days (IQR: 4–7; range: 1–74) (Figure 3).

Clients with both a *Chlamydia trachomatis* (Ct) and *Neisseria gonorrhoeae* (Ng) infection were included in both analyses.

Censored observations represent clients who did not return for treatment within 60 days (lost to follow-up).

For 20 consultations for both Ct and Ng, the date when clients returned to the clinic for treatment was unknown. They were therefore not included in the number at risk.

Missed treatment was observed in 4 of 155 consultations with clients positive for Ct (2.6%; 95% CI: 0.7–6.5) and in 5 of 158 consultations with clients positive for Ng (3.2%; 95% CI: 1.0–7.2).

## Discussion

We found that only presumptively treating formally notified, asymptomatic clients who are steady partners of index cases rather than treating all formally notified clients led to a substantial reduction in unnecessary antibiotic treatments. This reduction was observed consistently across all groups, including MSM, MSW and women, as well as across all age categories. Furthermore, following the policy change, clients still received treatment within a median of 6–7 days and fewer than 3% of clients with an infection did not return for treatment.

Before the policy change, the proportion of consultations in which presumptive antibiotic treatment was offered to notified clients who were partners of persons with Ct or Ng infection that later tested negative was 59.6% and 65.3%, respectively. These proportions align with findings from previous studies, which report that in 59.3–68.6% of clients who received a partner notification followed by presumptive treatment, a Ct or Ng infection was later ruled out [21-23]. This shows that the majority of clients who received presumptive treatment had no need for antibiotics. This was a key reason for CSH Amsterdam to restrict presumptive treatment for Ct or Ng to clients notified by steady partners only. Indeed, after the policy change, the proportion of consultations in which notified clients had received presumptive treatment but tested negative for Ct and Ng decreased significantly. Similar results were observed in a study at the Melbourne Sexual Health Centre in Australia, where the proportion of clients who tested negative for Ng, but received ceftriaxone presumptively decreased from 74.8% (92/123) to 60.6% (143/236) after the policy was changed from presumptively treating all clients with a partner notification for Ng to no presumptive treatment at all for clients with an Ng notification [24]. The Dutch policy change, which limits presumptive treatment to notifications from steady partners, lies between the less restrictive US Centers for Disease Control and Prevention guidelines (presumptive treatment of all recent partners within 60 days) and the more restrictive International Union against Sexually Transmitted Infections (IUSTI) guidance, which generally prioritises testing first [12,13]. The reduction in unnecessary antibiotic treatment we observed supports a more selective approach, consistent with the Australian guidelines that only consider presumptive treatment after very recent (i.e. in the past 2 weeks) exposure [14,15].

Following the policy change, we found that most clients with a Ct or Ng infection who had not received presumptive treatment returned for treatment within a week. This finding also aligns with results from the Melbourne Sexual Health Centre, where nearly 90% of clients returned for treatment after presumptive treatment for Ng was discontinued [24]. Concerns about a substantial treatment delay were therefore not supported in that setting. The high proportion of return to CSH Amsterdam is likely supported by free testing and treatment services combined with multi-channel reminders (text message, email and phone). Prior studies show that text notifications can reduce time to treatment and missed returns [25]. However, the time period between the initial test and antibiotic administration should ideally be shortened to prevent onward transmission.

Our study observed a significant increase in the proportion of notified steady partners of an index case following the policy change on 1 March 2020 (an increase of ca 19 percentage points), while notifications of non-steady partners of an index case concomitantly declined. A possible explanation could be the increased awareness among clients under the new policy that presumptive treatment was only offered for steady partnerships. This may have prompted clients to self-declare as steady partners of the index case in order to obtain presumptive treatment. Another possible explanation is the onset of the COVID-19 pandemic around the same time as the policy change on 1 March 2020, along with the associated government-imposed social restrictions. These restrictions not only advised limiting the number of non-steady partners but also encouraged clients to engage in sexual activity exclusively within steady relationships [26]. This public health messaging may have influenced both sexual behaviour and partner notification patterns, contributing to the observed shift.

It is well established that excessive and unnecessary antibiotic treatment contributes to the overall rise in AMR [27,28]. Additionally, the European Medicines Agency reaffirmed the urgency of reducing antibiotic treatment [28]. This underscores the continued importance of antibiotic stewardship and the need to explore innovative strategies for minimising antibiotic treatment without compromising public health or an individual’s quality of life. Recently, presumptive treatment was restricted further to only very recent exposures (i.e. 2 weeks) at all CSH in the Netherlands after a policy change on 19 February 2025 [18,19]. Additionally, to qualify for presumptive treatment for Ct, the index case must have had symptoms. This additional requirement results from the ongoing debate over screening asymptomatic clients for Ct. Some countries, including the Netherlands, have discontinued Ct screening of asymptomatic people in view of the low risk of sequelae of asymptomatic Ct infections [29,30]. This development reflects ongoing efforts to minimise unnecessary antibiotic treatment. The potential impact of the change implemented on 19 February 2025 on further reducing unnecessary antibiotic treatment needs to be investigated.

This study is not without limitations. First, there could be an underestimation of the proportion of clients who received unnecessary antibiotic treatment since Ct and Ng infections may clear spontaneously [31,32]. Spontaneous clearance of asymptomatic Ng infections has been reported in ca 15–30% of cases within 1–2 weeks [31-33]. In addition, Ct infections may also clear spontaneously within weeks [31,34]. Second, some clients who tested positive for Ct or Ng and who did not receive presumptive treatment at CSH Amsterdam may have received treatment elsewhere. However, this was likely a very low number. Third, with 67% of the included clients being MSM, the generalisability to other populations, such as MSW and women, is limited. Fourth, partner notification consultations at CSH Amsterdam continued throughout the COVID-19 pandemic [35]. Nevertheless, indirect effects of the pandemic (such as lockdown measures and public health advice to limit contacts or to have sex primarily with a steady partner) might have influenced consultation patterns and partner notification behaviours. Because the policy change coincided with the onset of the pandemic, residual temporal confounding cannot be excluded. However, results were largely similar when adjusting for calendar time. Fifth, although informally notified partners constituted the vast majority of partner notifications in routine practice, they follow a different clinical pathway, which remained unchanged under the new policy. Therefore, our findings are most applicable to settings where presumptive treatment is only considered for formally notified sexual partners. Sixth, we have no data on the time between receipt of partner notification (or last sexual exposure) and presentation for testing at CSH Amsterdam. If testing is performed too soon, some infections may be missed thereby overestimating the number of uninfected clients. While a positive NAAT may occur as early as 1 to 3 days post exposure, repeat testing may be needed if the first test was performed less than 2 weeks after exposure [17]. Last, we could not account for informal use of doxycycline post-exposure prophylaxis (doxyPEP), which has been reported among MSM and transgender and gender diverse people in the Netherlands [36]. Currently, doxyPEP is not recommended or prescribed by CSH in the Netherlands [37] and data on informal use were not routinely collected at CSH Amsterdam until 1 January 2025. Informal doxyPEP use may have influenced STI incidence and background antibiotic exposure, albeit an earlier study from the Amsterdam Cohort Studies suggests that doxyPEP use was low between October 2021 and October 2022 (2.5% of 593 MSM) [38].

## Conclusions

The change in policy regarding reducing presumptive treatment in consultations with partner notifications for Ct or Ng infections resulted in a substantial reduction in unnecessary antibiotic treatments. Furthermore, the proportion of follow-up treatment in clients with a positive Ct or Ng result, when presumptive treatment had not been offered, was notably high. Given the global concerns surrounding AMR and the international call for antimicrobial stewardship, these findings support more selective partner management strategies as a positive step within broader European antimicrobial stewardship efforts. However, the median time to treatment of 7 days for Ct and 6 days for Ng should ideally be shortened. Future efforts should focus on further reducing unnecessary antibiotic treatment as part of antibiotic stewardship, without compromising public health or the quality of life of clients.

## Data Availability

Data are available upon reasonable request. On reasonable request to the last author (h.j.devries@amsterdamumc.nl), the following data will be made available after publication: de-identified participant data. Data will be shared after approval of an analysis proposal by the co-authors (BT, MSvdL, LD, JS, EH, VWJ, HdV).

## Supplementary Data

382000 Supplementary Material
