# Supplementary material for: Evaluating a new partner management strategy for bacterial sexually transmitted infections after a change in 2020 in Amsterdam, the Netherlands, 2017 to 2023
Source: Euro Surveill. 2026 Jun 4;31(22):2500754. doi: 10.2807/1560-7917.ES.2026.31.22.2500754 (PMC13241789; doi:10.2807/1560-7917.ES.2026.31.22.2500754)
Supplement: Supplementary Material [file 25-00754_JONGEN_Supplement.pdf]

This supplementary material is hosted by *Eurosurveillance* as supporting information alongside the article ‘Evaluating a new partner management strategy for bacterial sexually transmitted infections after a change in 2020 in Amsterdam, the Netherlands, 2017 to 2023’, on behalf of the authors, who remain responsible for the accuracy and appropriateness of the content. The same standards for ethics, copyright, attributions and permissions as for the article apply. Supplements are not edited by *Eurosurveillance* and the journal is not responsible for the maintenance of any links or email addresses provided therein.

Table of contents

Supplementary Figure S1: Flowchart of the partner management policies before and after 1 March 2020 in partner notifications for *Chlamydia trachomatis* and *Neisseria gonorrhoeae*, Centre for Sexual Health, Amsterdam, the Netherlands, 1 March 2017 to 1 March 2023 3

Supplementary Table S1: Sensitivity analyses of change in unnecessary antibiotic use in partner notification consultations for *Chlamydia trachomatis* infections, Centre for Sexual Health, Amsterdam, the Netherlands, 1 March 2017 to 1 March 2023 4

Supplementary Table S2: Sensitivity analyses of change in unnecessary antibiotic use in partner notification consultations for *Neisseria gonorrhoeae* infections, Centre for Sexual Health, Amsterdam, the Netherlands, 1 March 2017 to 1 March 2023 6

Supplementary Figure S1: Flowchart of the partner management policies before and after 1 March 2020 in partner notifications for *Chlamydia trachomatis* and *Neisseria gonorrhoeae*, Centre for Sexual Health, Amsterdam, the Netherlands, 1 March 2017 to 1 March 2023

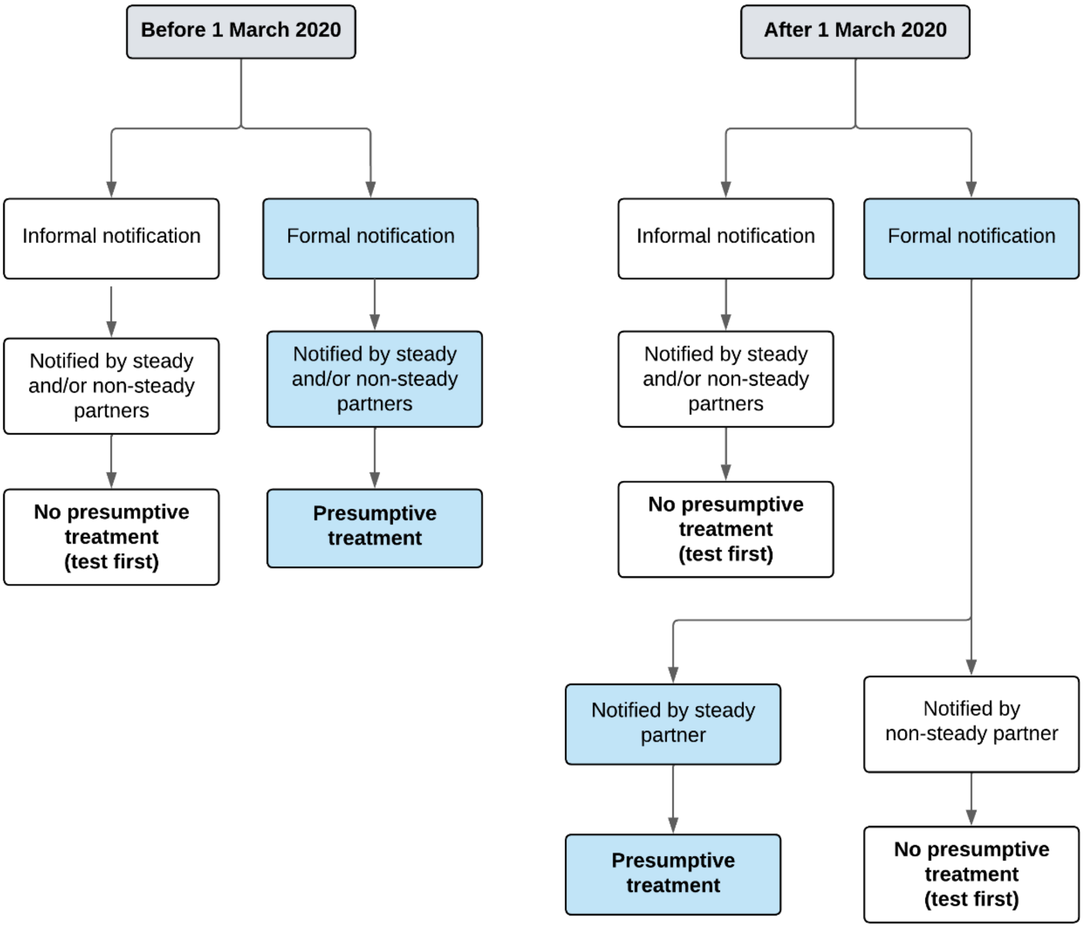

Note: A steady partnership is one where the notified client: (1) had sex with the index in the previous 2 months, and (2) intents to have sex again with the index, non-steady partnership are all other sexual engagements.

Supplementary Table S1: Sensitivity analyses of change in unnecessary antibiotic use in partner notification consultations for *Chlamydia trachomatis* infections, Centre for Sexual Health, Amsterdam, the Netherlands, 1 March 2017 to 1 March 2023

	**No. of unnecessary antibiotic use**	**Unnecessary antibiotic use Univariable relative risk regression**			**Unnecessary antibiotic use Multivariable relative risk regression**		
	n / N (%)	*Crude RR*	*95% CI*	*p-value*[1]	*aRR*	*95% CI*	*p-value*[1]
Gender and sexual behaviour[2,3]							
Men who have sex with men							
**Policy**							
Old	488 / 665 (73.4%)	REF			REF		
New	215 / 860 (25.0%)	0.34	0.30-0.39	<0.001	0.35	0.30-0.40	<0.001
Men who exclusively have sex with women[5]							
**Policy**							
Old	226 / 458 (49.3%)	REF			REF		
New	83 / 267 (31.1%)	0.63	0.52-0.77	<0.001	0.61	0.50-0.75	<0.001
Women[4]							
**Policy**							
Old	72 / 194 (37.1%)	REF			REF		
New	25 / 145 (17.2%)	0.46	0.31-0.69	<0.001	0.46	0.31-0.68	<0.001
Age groups[5]							
18-24 years old							
**Policy**							
Old	192 / 436 (44.0%)	REF			REF		
New	66 / 331 (19.9%)	0.45	0.36-0.58	<0.001	0.44	0.34-0.57	<0.001
25-29 years old							
**Policy**							
Old	188 / 307 (61.2%)	REF			REF		
New	62 / 280 (22.1%)	0.36	0.28-0.46	<0.001	0.37	0.29-0.48	<0.001
30-34 years old							
**Policy**							
Old	131 / 203 (64.5%)	REF			REF		
New	53 / 225 (23.6%)	0.37	0.28-0.47	<0.001	0.35	0.26-0.48	<0.001

Supplementary Table 1. (continued)

	No. of unnecessary antibiotic use	Unnecessary antibiotic use Univariable relative risk regression			Unnecessary antibiotic use Multivariable relative risk regression		
	n / N (%)	*Crude RR*	*95% CI*	*p-value*[1]	*aRR*	*95% CI*	*p-value*[1]
35 years old or older							
**Policy**							
Old	275 / 372 (72.9%)	REF			REF		
New	143 / 439 (32.6%)	0.44	0.38-0.51	<0.001	0.46	0.38-0.55	<0.001

Abbreviations: aRR, adjusted relative risk; CI, confidence interval; REF, reference, RR, relative risk.

1. P-values were derived from relative risk regression (i.e., generalized estimating equations with a Poisson distribution, log link, and robust variance estimation) to account for repeated consultations per individual, unless otherwise stated.
2. Transgender and gender diverse people were not analysed due to low numbers.
3. Adjusted relative risks were adjusted for age, country of birth, highest education level, number of sexual partners in the last 6 months, HIV status and HIV PrEP use, and self-reported history of an STI in the last 6 months.
4. Adjusted relative risks were adjusted for age, country of birth, highest education level, number of sexual partners in the last 6 months, and self-reported history of an STI in the last 6 months.
5. Adjusted relative risks were adjusted for gender and sexual behaviour (transgender and gender diverse people excluded due to low numbers), country of birth, highest education level, number of sexual partners in the last 6 months, HIV status and HIV PrEP use, and self-reported history of an STI in the last 6 months.

Note: The old policy refers to the period before 1 March 2020, whereas the new policy refers to the period from 1 March 2020.

Supplementary Table S2: Sensitivity analyses of change in unnecessary antibiotic use in partner notification consultations for *Neisseria gonorrhoeae* infections, Centre for Sexual Health, Amsterdam, the Netherlands, 1 March 2017 to 1 March 2023

	**No. of unnecessary antibiotic use**	**Unnecessary antibiotic use Univariable relative risk regression**			**Unnecessary antibiotic use Multivariable relative risk regression**		
	n / N (%)	*Crude RR*	*95% CI*	*p-value[1]*	*aRR*	*95% CI*	*p-value[1]*
Gender and sexual behaviour[2,3]							
Men who have sex with men							
**Policy**							
Old	670 / 1,027 (65.2%)	REF			REF		
New	321 / 1,298 (24.7%)	0.38	0.34-0.42	<0.001	0.37	0.32-0.41	<0.001
Men who exclusively have sex with women[5]							
**Policy**							
Old	40 / 52 (76.9%)	REF			REF		
New	12 / 35 (34.3%)	0.45	0.28-0.74	0.001	0.39	0.24-0.64	<0.001
Women[4]							
**Policy**							
Old	18 / 36 (50.0%)	REF			REF		
New	7 / 28 (25.0%)	0.50	0.24-1.03	0.061	0.43	0.20-0.95	0.036
Age groups[5]							
18-24 years old							
**Policy**							
Old	89 / 174 (51.2%)	REF			REF		
New	21 / 135 (15.6%)	0.31	0.20-0.46	<0.001	0.29	0.19-0.46	<0.001
25-29 years old							
**Policy**							
Old	156 / 258 (60.5%)	REF			REF		
New	56 / 268 (20.9%)	0.35	0.27-0.46	<0.001	0.38	0.28-0.51	<0.001
30-34 years old							
**Policy**							
Old	140 / 205 (68.3%)	REF			REF		
New	85 / 335 (25.4%)	0.37	0.30-0.45	<0.001	0.35	0.27-0.45	<0.001

Supplementary Table 2. (continued)

	No. of unnecessary antibiotic use	Unnecessary antibiotic use Univariable relative risk regression			Unnecessary antibiotic use Multivariable relative risk regression		
	n / N (%)	*Crude RR*	*95% CI*	*p-value*[1]	*aRR*	*95% CI*	*p-value*[1]
35 years old or older							
**Policy**							
Old	344 / 480 (71.7%)	REF			REF		
New	179 / 631 (28.4%)	0.40	0.35-0.46	<0.001	0.40	0.34-0.47	<0.001

Abbreviations: aRR, adjusted relative risk; CI, confidence interval; REF, reference, RR, relative risk.

1. P-values were derived from relative risk regression (i.e., generalized estimating equations with a Poisson distribution, log link, and robust variance estimation) to account for repeated consultations per individual, unless otherwise stated.
2. Transgender and gender diverse people were not analysed due to low numbers.
3. Adjusted relative risks were adjusted for age, country of birth, highest education level, number of sexual partners in the last 6 months, HIV status and HIV PrEP use, and self-reported history of an STI in the last 6 months.
4. Adjusted relative risks were adjusted for age, country of birth, highest education level, number of sexual partners in the last 6 months, and self-reported history of an STI in the last 6 months.
5. Adjusted relative risks were adjusted for gender and sexual behaviour (transgender and gender diverse people excluded due to low numbers), country of birth, highest education level, number of sexual partners in the last 6 months, HIV status and HIV PrEP use, and self-reported history of an STI in the last 6 months.

Note: The old policy refers to the period before 1 March 2020, whereas the new policy refers to the period from 1 March 2020.
